# On Small-Pox Succeeding Vaccination, at Crediton

**Published:** 1814-12

**Authors:** Thomas Hugo

**Affiliations:** Crediton


*For the Medical and Physical Journal.*

### *On Small-pox succeeding Vaccination, at Crediton;* by Mr. Thomas Hugo.

THE small-pox has prevailed very generally in this neighbourhood for several months, the first case having occurred about the month of April last. The strong bias which existed in the public opinion against vaccination, in consequence of several reported failures, and the preference which was therefore given to the *variolous* inoculation, was, as usual, the means of a rapid diffusion of its contagion among the poorer inhabitants, most of whom refused the gratuitous offer of vaccination which was made them. The character of the variolous disease, during the summer months, was more than commonly severe; and the number of its victims exceeded, I believe, the usual proportion.

This general prevalence of small-pox has necessarily exposed to a strong infection a large proportion of those who had been previously vaccinated, and has put the security afforded by vaccination to a severer test than at any other time since its introduction into this neighbourhood. I regret to state, that its security against the variolous contagion has been found *here* to be less complete than I was induced to expect, not only from a consideration of the concurrent public testimony in its favour, but also from the unequivocal proofs I had myself so frequently witnessed in support of the same opinion.

As soon as the variolous infection had extended in the town, instances of small-pox began to occur among persons who had been vaccinated. These, it was at first considered, were occasional deviations only, from which the small-pox itself is not exempt; or that it might have happened in cases where the vaccine infection had been confined merely to the inoculated part, especially as, before this time, I had not observed the occurrence of small-pox in a single case where the *test* of a second vaccination had satisfactorily proved a constitutional affection. The cases of failure became, however, at length, so numerous and decisive, that they could not fail to excite alarm, and to engage the serious attention of the medical practitioners.

Twenty-five persons, who, from the regular progress of the vaccine vesicles, were considered as secure against the small-pox, have casually received the variolous infection, during the last six months. I allude to those cases only which have been attended by medical practitioners, and where the evidence was considered, in all respects, as conclusive.

Many

Many others occurred which were thought doubtful, and some were reported by persons not of the medical profession, and therefore not entitled to implicit credit. Several vaccinated children also, on exposure to the variolous contagion, though it was succeeded by *no eruption*, were as much indisposed, and with exactly the same symptoms, as those were who had a decided eruption of small-pox.

With the exception of two cases, all the above were persons who had been vaccinated upwards of six years. The *most* numerous eruption was in some of those where the longest period had elapsed since vaccination; but it can scarcely be supposed to depend *wholly* on this circumstance, since many of the first vaccinated are found to be still secure. And, *in general*, the number of the eruption, and the severity of the previous symptoms, were as great in the later as in the earlier cases.

In few instances did the number of the eruption bear any proportion to the severity of the eruptive fever. The fever in its attack and progress was commonly violent. The heat was excessive, the pulse very quick, universal languor, pain in the head and loins, frequent vomiting, occasional delirium in the night, and sometimes convulsions. These symptoms, after having occasioned considerable alarm for three or four days, were succeeded by a distinct and mild eruption, which dissipated all apprehension of danger. In some cases, where the eruption was numerous, it suppurated as usual, but it was more commonly hard and tubercular, with little inflammation, and the suppuration very imperfect, the patient almost always passing through this stage of the disorder with scarcely any indisposition.

In many families, consisting of several children, who were considered as having regularly passed through the vaccine disease, it has happened that one or two only were susceptible of the small-pox; but in one instance, *four* children out of five in the same family, who had been vaccinated at different times and by different practitioners, successively had the variolous fever and eruption.

To obviate the possibility of doubt that the disorder I have described was really variolous, I inoculated with matter taken from some of the pustules, and produced the genuine small-pox. I have also witnessed a case of *confluent* small-pox, where the infection was undoubtedly derived from a person who had the disease after vaccination.

The above facts may, I presume, be regarded as satisfactory evidence that in a variety of instances, vaccination affords a partial security only against the contagion of the small-pox. What the proportion of these cases is to the total total number of persons vaccinated, it is difficult to ascertain, but, from the number which have occurred here, in the space of six months, it is evidently larger than might have been expected. I believe that vaccination has no where been practised with more scrupulous attention to the characteric appearance of the vesicle; and I have in no case which had been entrusted to my own care, neglected to ascertain the constitutional affection, by the test of a second vaccination. It is impossible, I conceive, therefore, to explain these unsuccessful cases on the supposition that the preceding vaccination had been spurious and irregular. Future observation and experience may possibly explain on what circumstance this uncertainty depends.

That the small-pox, during the late epidemic, has been particularly severe and virulent, I have before noticed. Probably the milder cases of cow-pox may not have excited sufficient action in the system to withstand wholly so active and concentrated a contagion. In two instances, even the small-pox itself afforded no security. In the one, the person had been inoculated thirty years before: it was recollected that the febrile symptoms ran high, and that he had an universal rash, which was considered as variolous, and the marks of the inoculation on both the arms are still very large, but the extent of the eruption is not remembered. This person, though he had since been frequently exposed to the infection of the small-pox, received at this time the contagion, and had a very full eruption. In the other case, the person was inoculated about eight years since, and had then many eruptions, yet he had now the disease confluently. In both these instances, the secondary fever appeared to have been considerably restrained by the previous disease.

Notwithstanding vaccination has afforded in so many instances an imperfect security only against the variolous contagion, still its credit continues undiminished as a security against the *danger* of the small-pox. In those cases where the fever was most severe, all apprehension of danger ceased on the appearance of the eruption; and, amidst the mortality of the small-pox, the vaccinated persons, if infected, were secure. This circumstance naturally diminished the alarm which was at first felt, and those who had passed the vaccine disease, were therefore not very solicitous to be inoculated with variolous matter. The appearances, however, produced by inoculation were similar to those occasioned by the casual infection; a great majority were wholly secure; in some it excited fever, and often a rash evidently variolous, but without the pustular eruption; and, in a few instances, a small and imperfect eruption succeeded, which partially suppurated.

This constant effect of vaccination, in producing a mild and distinct small-pox, gained it many proselytes among the poorer inhabitants, who frequently requested their children might be vaccinated, not with the view of preventing the small-pox altogether, but for the purpose of depriving it of its danger; and this, it must be acknowledged, is not an incorrect idea of the subject.

In the cases which have occasioned the preceding observations, nothing was remarked in the appearance of the vesicles on the part inoculated which could lead to a suspicion of their insufficiency; nor does it appear that the *manner* of vaccinating was uniform, some practitioners having trusted to *one* puncture, whilst others employed *two*, either at the same time, or by instituting the second, by way of test, a few days afterwards. It is probable that the vaccination afforded, even in these instances, a temporary security. It is seldom practised in the country except during the prevalence of the small-pox, and there has been no instance of small-pox succeeding vaccination, even though surrounded by the disease, till after the lapse of some time, which probably varies according to the peculiar constitution of the person, and the strength of the contagion.

The foregoing remarks are made solely with the view of exciting the attention of the profession in similar cases, to detect, if possible, the particular circumstances on which this partial security depends. The blessing which it is hoped will result from vaccination, the annihilation of the small-pox, must, in a great measure, rest on it. With all its present imperfections, it must still be allowed that its powers, and indeed its advantages, are great; and humanity can scarcely be better employed than in endeavouring to render those advantages more general, and to realize the expectations which were originally formed of it.

*Crediton; Oct.* 25, 1814. THOMAS HUGO.

